# PRERISK Study: A Randomized Controlled Trial Evaluating a sFlt-1/PlGF-Based Calculator for Preeclampsia Hospitalization

**DOI:** 10.1161/HYPERTENSIONAHA.124.24386

**Published:** 2025-04-17

**Authors:** Anna C.M. Kluivers, Rugina I. Neuman, Langeza Saleh, Henk Russcher, Ingrid A. Brussé, Jerome M.J. Cornette, Eric A.P. Steegers, Marijke C. van der Weide, Joris van Drongelen, Ralph R. Scholten, Antonius E. van Herwaarden, Sanne J. Gordijn, Anneke C. Muller Kobold, Wessel Ganzevoort, Sharon M. Wesselius, Maurits C.F.J. de Rotte, Robert Aardenburg, Maarten Raijmakers, Willy Visser, A.H. Jan Danser

**Affiliations:** 1 Department of Internal Medicine, Division of Pharmacology and Vascular Medicine, Erasmus MC, University Medical Center, Rotterdam, the Netherlands (A.C.M. Kluivers, R.I.N., W.V., A.H.J.D.).; 2 Department of Obstetrics and Gynecology, Division Obstetrics and Fetal Medicine, Erasmus MC, University Medical Center, Rotterdam, the Netherlands (A.C.M. Kluivers, L.S., I.A.B., J.M.J.C., E.A.P.S.).; 3 Department of Clinical Chemistry, Erasmus MC, University Medical Center, Rotterdam, the Netherlands (H.R.).; 4 Department of Obstetrics and Gynecology, Amsterdam University Medical Center, University of Amsterdam, the Netherlands (M.C.W., W.G., S.M.W.).; 5 Department of Obstetrics and Gynecology, Radboud University Medical Center, Nijmegen, the Netherlands (J.D., R.R.S.).; 6 Department of Clinical Chemistry, Radboud University Medical Center, Nijmegen, the Netherlands (A.E.H.).; 7 Department of Obstetrics and Gynecology, University of Groningen,University Medical Center Groningen, Groningen, the Netherlands (S.J.G.).; 8 Department of Laboratory medicine, University of Groningen, University Medical Center Groningen, the Netherlands (A.C. Muller Kobold).; 9 Laboratory of Specialized Diagnostics and Research, Department of Laboratory medicine, Amsterdam University Medical Center, University of Amsterdam, the Netherlands (M.C.F.J.R.).; 10 Department of Obstetrics and Gynecology, Zuyderland Medical Center, Heerlen, The Netherlands (R.A.).; 11 Department of Clinical Chemistry, Zuyderland Medical Center, Heerlen, the Netherlands (M.R.).

**Keywords:** pre-eclampsia, placental growth factor, pregnancy, risk assessment, soluble fms-like tyrosine kinase-1

## Abstract

**BACKGROUND:**

A model based on the soluble Fms-like tyrosine kinase-1/placental growth factor ratio, gestational age, and the urinary protein-to-creatinine ratio (PRERISK calculator) has been developed to predict preeclampsia-related maternal-fetal complications. Here, we tested whether this model can reduce hospital admissions without increasing complication rates among women with suspected or confirmed preeclampsia.

**METHODS:**

In this multicenter, open-label, randomized controlled trial conducted at 5 Dutch medical centers, women with suspected or confirmed preeclampsia were randomly assigned to the intervention group, where admission was guided by the PRERISK score using a 5% cutoff, or to the control group receiving routine care with a concealed PRERISK score. Two co-primary outcomes were the incidence of maternal-fetal preeclampsia-related complications (noninferiority) and the proportion of women with a hospitalization ratio (=admission days/inclusion days) ≤0.05 (superiority).

**RESULTS:**

The intervention and control groups included 442 and 435 women, respectively. In the intention-to-treat analysis, complications occurred in 41.6% of the intervention group versus 39.5% of the control group (adjusted relative risk 1.06 [95% CI, 0.92–1.22]; *P*=0.43). The proportion of women achieving a hospitalization ratio ≤0.05 was 23.6% in the intervention group and 26.3% in the control group (adjusted relative risk, 0.89 [95% CI, 0.71–1.13]; *P*=0.34). The latter was comparable in the per-protocol analysis (adjusted relative risk, 0.87 [95% CI, 0.64–1.19]; *P*=0.38), while in this analysis, complications occurred in 47.8% of the intervention group (n=251) versus 41.7% of the control group (n=365; adjusted relative risk 1.19 [95% CI, 1.03–1.38]; *P*=0.02).

**CONCLUSIONS:**

Routine screening with the PRERISK score and a 5% cutoff in patients with suspected or confirmed preeclampsia does not decrease hospitalization and is therefore not recommended.

**REGISTRATION:**

URL: https://onderzoekmetmensen.nl/nl/trial/48687; Unique identifier: NL63386.078.17, NL-OMON48687.

**NOVELTY AND RELEVANCE**

**What Is New?**

Our multicenter, randomized controlled trial evaluated the PRERISK calculator, a model incorporating the soluble Fms-like tyrosine kinase-1/proangiogenic placental growth factor ratio, gestational age and the urinary protein-to-creatinine ratio, as a tool to minimize the number of hospital admission days for patients with suspected or diagnosed preeclampsia without increasing the amount of maternal and fetal complications.

**What Is Relevant?**

We found that using the PRERISK score in patients with (suspected) preeclampsia did not reduce the duration of hospitalization in these patients, while the rate of preeclampsia-related complications was similar in both groups.

**Clinical/Pathophysiological Implications?**

The effectiveness of the soluble Fms-like tyrosine kinase-1/proangiogenic placental growth factor ratio as a tool for the diagnosis of preeclampsia has been elaborately assessed. Our study now adds that using the soluble Fms-like tyrosine kinase-1/proangiogenic placental growth factor ratio-based PRERISK calculator does not decrease the hospitalization in patients with suspected or confirmed preeclampsia. Considering increasing health care demands and costs, implementation of a new diagnostic test should be carefully considered. Based on our findings, we do not recommend routine screening using the PRERISK calculator in women with suspected or confirmed preeclampsia.

Preeclampsia is a pregnancy disorder that contributes to maternal and perinatal morbidity and mortality.^1–3^ The definition of preeclampsia varies, from a research definition of hypertension with proteinuria after 20 weeks of gestation to a broader clinical definition involving maternal organ failure and fetal growth restriction, reflecting its variable clinical phenotype.^1,2,4^ The heterogenous presentation and clinical course of preeclampsia limit the ability to accurately predict the occurrence of associated adverse events, although several prediction models have been developed.^4–8^ While expectant management with pregnancy prolongation at preterm gestational ages can be achieved in some women, others require prompt delivery.^9,10^ Consequently, management varies among clinicians, and most patients diagnosed with preeclampsia are hospitalized until birth.^10,11^ Recent developments in home monitoring offer the opportunity to perform this in an outpatient setting.^12^ However, accurate prediction of preeclampsia-related complications continues to be an unmet need.

Abnormal placentation with insufficient maternal spiral artery remodeling is considered the predominant underlying mechanism of preeclampsia.^1^ The ensuing placental hypoxia leads to the release of the antiangiogenic soluble Fms-like tyrosine kinase-1 (sFlt-1) from the placenta into the maternal circulation, subsequently binding the proangiogenic placental growth factor (PlGF), thus increasing the sFlt-1/PlGF ratio.^1,13^ This antiangiogenic state may underlie the endothelial inflammation and dysfunction that explains the clinical spectrum. The sFlt-1/PlGF ratio has been proposed as a biomarker to predict the occurrence of preeclampsia and its complications, using the cutoff points <38 (rule out) and >85 (rule in).^13–15^ Yet, sFlt-1, PlGF and their ratio vary during pregnancy, implying that the cutoff points also differ according to gestational age.^10^ We, therefore, developed a model (the PRERISK calculator) to predict preeclampsia-related maternal and fetal complications based on the sFlt-1/PlGF ratio as a continuous variable and incorporating gestational age and the urinary protein-to-creatinine ratio at the time of blood sampling.^10,11^ It allows the accurate prediction of a preeclampsia-related maternal and fetal composite outcome within 7, 14, and 30 days in women with (suspected) preeclampsia and has been internally validated.^11^

We hypothesized that the application of the PRERISK calculator can safely reduce the duration of hospitalization in patients with suspected or confirmed preeclampsia, without increasing complications. In the present study, we prospectively investigated the application of the PRERISK score. For this, we evaluated the PRERISK score on a weekly basis in a randomized controlled trial, in patients with (suspected) preeclampsia, that is, the same population on which the calculator was based.

## Methods

### Data Availability

The data that support the findings of this study are available from the corresponding author upon reasonable request.

### Study Design

The PRERISK study was an investigator-initiated, multicenter, open-label, randomized controlled trial with both superiority and noninferiority elements, using a co-primary outcome. The study was conducted at 5 centers in the Netherlands (Table S1) as part of the Dutch Consortium for Healthcare Evaluation and Research in Obstetrics and Gynaecology, from August 2019 to July 2024. The protocol was approved by the Medical Research Ethics Committee of Erasmus University Medical Center (MEC-2018-003) and accepted by the boards of the other participating centers. The trial was registered in the Dutch Trial Register (NL-OMON48687). The trial adhered to the Guidelines for Good Clinical Practice, and the authors assume responsibility for the accuracy and completeness of the data and analyses, as well as for the fidelity of the trial and report to the protocol. The CONSORT (Consolidated Standards of Reporting Trials) was applied as reporting guideline.^16^ An independent Data Safety Monitoring Board of the Dutch Society of Obstetrics and Gynecology conducted an interim safety analysis after the inclusion of 25% and 50% of the intended number of participants.

### Participants

Women aged ≥18 years, with a vital, singleton pregnancy (20^+0^ to 36^+^6 weeks of gestation at the time of inclusion) with (suspected) preeclampsia according to predefined criteria, were eligible for participation. Suspected preeclampsia was defined as new onset of hypertension (systolic blood pressure ≥140 mm Hg or diastolic blood pressure ≥90 mm Hg), aggravation of preexisting hypertension (systolic or diastolic blood pressure increase ≥10 mm Hg or increased need for antihypertensive medication), new onset of proteinuria (protein-to-creatinine ratio ≥30 mg/mmol; 24-hour urine protein ≥0.3 g/24 h; dipstick ≥2+ protein), aggravation of preexisting proteinuria (increase of ≥50% in protein-to-creatinine ratio compared with the last measurement), or clinical signs suggestive of preeclampsia (eg, excessive edema or severe swelling in the face, hands, and feet or epigastric pain).^17^ Confirmed preeclampsia was defined as new onset or aggravation of preexisting hypertension and new onset or aggravation of preexisting proteinuria. Patients were excluded if they were admitted to the hospital with another reason than preeclampsia at the time of inclusion, if they had hemolysis, elevated liver enzymes and low platelets syndrome (lactate dehydrogenase >321 U/L; haptoglobin <0.10 g/L; alanine transaminase >41 U/L; platelet count <100×10^9^/L), or partial hemolysis, elevated liver enzymes and low platelets syndrome (>2 of the aforementioned criteria) at the time of inclusion, if they had intrauterine fetal demise at inclusion, if they were pregnant of a fetus affected by major congenital birth defects or chromosomal abnormalities, or if they were unable to provide written informed consent. All participants provided written informed consent.

### Randomization and Masking

Participants were randomly assigned in a 1:1 ratio to either the intervention group (revealed PRERISK score to the clinician, patient, and researchers, with management based upon the PRERISK score) or the control group (concealed PRERISK score for clinician, patient, and researchers, with routine clinical care), stratified by center and gestational age (<34 weeks and ≥34 weeks). Randomization was performed using a secure Web-based server (Castor Electronic Data Capture [EDC]) in variable permuted blocks, with block sizes of 4 and 6.

### Procedures

To determine the PRERISK score, the gestational age at blood sampling in days, the sFlt-1/PlGF ratio,^18^ and the urinary protein-to-creatinine ratio were incorporated into a risk chart^10,11^ (Table S2; Figure S1) embedded in the patient’s electronic health record. For patients in the intervention group, the result of the PRERISK score was reported within 2 hours. The sFlt-1/PlGF ratio was not revealed. In both study groups, the protein-to-creatinine ratio was only revealed if the clinician specifically requested this measurement as part of the patient work-up or follow-up.

In the intervention group, participants remained in outpatient care when the PRERISK score was <5.0% or hospitalized when the PRERISK score was ≥5.0%. Home blood pressure monitoring was considered as outpatient follow-up. The cutoff point of 5.0% was established based on expert opinion and its high sensitivity (98%) ruling out an adverse outcome within 1 week.^10^ The PRERISK score was repeated weekly until birth, guiding patient management based on the most recent PRERISK score. Clinicians were strongly advised to adhere to the study protocol, but were permitted to deviate from the protocol, when deemed necessary on clinical grounds. In the control group, weekly blood samples were collected, but the PRERISK score remained concealed until analysis. These patients received routine clinical care, which entailed admission with a confirmed diagnosis of preeclampsia or outpatient follow-up in case preeclampsia was not confirmed (except when intravenous treatment was required due to severe hypertension in the absence of preeclampsia). Uniformity was promoted by repeated educations presentations at the participating sites and through local principal investigators.

### Outcomes

The study had 2 co-primary outcomes. The first co-primary outcome was a composite of preeclampsia-related maternal and fetal complications, evaluated in a noninferiority analysis. Complications were defined as acute kidney injury, cerebral complications, eclampsia, hemolysis, elevated liver enzymes and low platelets syndrome, pulmonary edema, placental abruption, visual disturbances (characterized by cortical blindness or retinal detachment), subcapsular liver hematoma, postpartum hemorrhage (>1000 mL blood loss after birth), fetal distress requiring expedited birth (Cesarean section), and fetal death. The second co-primary outcome was the proportion of patients with a hospitalization ratio ≤0.05, evaluated in a superiority analysis. The hospitalization ratio was introduced to the study protocol with the second amendment (October 18, 2019), following the advice of our trial methodologists. The hospitalization ratio reflects the number of admission days divided by the amount of inclusion days, involving a correction for the duration of inclusion as such. Admission is defined as 1 overnight stay. The hospitalization cutoff of 0.05 translates to 1 hospitalization day for 20 inclusion days.

Secondary outcomes were the number of outpatient clinical visits, gestational age at birth, days until birth, mode of birth, number of induced births, and the occurrence of neonatal complications (preterm birth <37 weeks of gestation, admission to neonatal intensive care, need for respiratory support, bronchopulmonary dysplasia, infant respiratory distress syndrome, necrotizing enterocolitis, sepsis, small-for-gestational age, cerebral complications, and neonatal death within 30 days after birth). As the 5% cutoff point was based on expert consensus, we additionally determined a new cutoff for the PRERISK score from the trial data after study completion to optimally rule out composite preeclampsia-related complications within 1 week, and compared it to the sFlt-1/PlGF ratio.

### Assessment of Biomarkers

Measurement of sFlt-1 and PlGF was performed at the clinical laboratory of each participating center, using the fully automated Elecsys assays for sFlt-1 and PlGF on an electrochemiluminescence immunoassay platform (Cobas 6000, e module; Roche Diagnostics, Mannheim, Germany). Data are expressed in picograms per milliliter.^19–22^ The maximum between-run coefficients of variation are 5.7% for the Elecsys sFlt-1 assay and 6.5% for the Elecsys PlGF assay. Controls were run on control material intended for this purpose. These variations did not influence PRERISK calculator results in such a way, that clinical decision-making was at stake. Before and during the study protocol, sFlt-1 and PlGF were not measured routinely at any of the participating sites. For the measurement of protein and creatinine, maternal mid-stream urine samples were collected. Measurement of protein and creatinine occurred at the clinical laboratory of each participating center with the Cobas 8000, c702 module (Roche Diagnostics). Between-day variation based on control material is 2.2% for protein and 2.0% for creatinine.

### Statistical Analysis

Regarding noninferiority, a 30% complication rate was expected in the control group.^11^ Based upon expert opinion and study feasibility, the noninferiority margin was set at a RR below 1.3 (comparable to a complication rate of <39% in the intervention group). At α=0.025 (1-sided) and β=80%, this yielded a sample size of 411 per study group. Second, pilot data in a comparable population indicated that 52.8% of the patients had a hospitalization ratio ≤0.05, which corresponds to 1 day of hospitalization per 20 inclusion days (1/20=0.05).^11^ To detect an absolute improvement of 10% (comparable to a RR of 1.19), a sample size of 379 per study group was required, at α=0.05 (2-sided) and β=80%. To study both hypotheses, we selected the largest sample size, and added 5% to correct for loss to follow-up, resulting in 432 patients per study group and a total of 864 participants.

The primary analysis was performed according to the intention-to-treat principle and was repeated in a per-protocol analysis, excluding patients whose management did not adhere to the study protocol. Protocol violations were described as admission for (suspected) preeclampsia or pregnancy-induced hypertension with a PRERISK score <5%; outpatient follow-up with a PRERISK score ≥5%, birth >7 days after the last PRERISK measurement, or no prepartum PRERISK measurements available. For the co-primary outcome, generalized linear regression analysis with a log link using the sandwich estimator for variance estimates was performed, and results are presented as crude RR with their associated 95% CIs and with correction for the stratification factors center and gestational age. The PRERISK strategy was defined to be noninferior to current practice when the upper bounds of the 95% CI of the RR of complications were <1.3. Both the intention-to-treat and per-protocol analyses needed to be noninferior for the hypothesis to be true. The PRERISK strategy was considered superior if leading to a significant RR of >1.19 regarding the hospitalization ratio ≤0.05 in the intervention group compared with the control group. Both noninferiority for complications and superiority for the proportion of patients with a hospitalization ratio ≤0.05 were required to conclude the effectiveness of the PRERISK strategy.

### Sensitivity Analysis

Two sensitivity analyses were conducted to correct for cases that were unjustly considered to have a hospitalization ratio of 0, due to first PRERISK calculator measurement, admission and delivery occurring on the same day. In the first, patients with first PRERISK calculator measurement, admission, and delivery on the same day were excluded. In the second sensitivity analysis, it was assumed that all patients at least had 1 inclusion day (the day of inclusion) and at least 1 admission day (the day of delivery), resulting in a start hospitalization ratio of 1, which might decrease depending on admission duration and inclusion duration. In this second analysis, we considered the hospitalization ratio as a continuous variable, rather than the proportion of patients with a hospitalization ratio ≤0.05, because patients would require a minimum of 20 inclusion days without any further admission days to get to a hospitalization ratio of 0.05.

Descriptive statistics are shown as mean (±SD) or median (interquartile range) for continuous variables and as number (percentage) for categorical variables. The normality of continuous variables was assessed visually. For the comparison of continuous variables between groups, an unpaired *t* test or Mann-Whitney *U* test (for skewed distribution) was performed. For the comparison of categorical variables between 2 groups, the χ^2^ test was applied. To evaluate the sensitivity and specificity of both the PRERISK score and the sFlt-1/PlGF ratio, a receiver operating characteristic curve was drawn using data from the control group. An optimal cutoff value for the PRERISK score was determined using the Youden statistic. As a post hoc analysis, the second co-primary outcome, that is, the proportion of women with a hospitalization ratio ≤0.05, was reassessed using the new optimal cutoff value.

All statistical analyses were performed with the use of IBM SPSS Statistics, version 28.0.1.0.

## Results

### Baseline Characteristics

**Figure 1.**
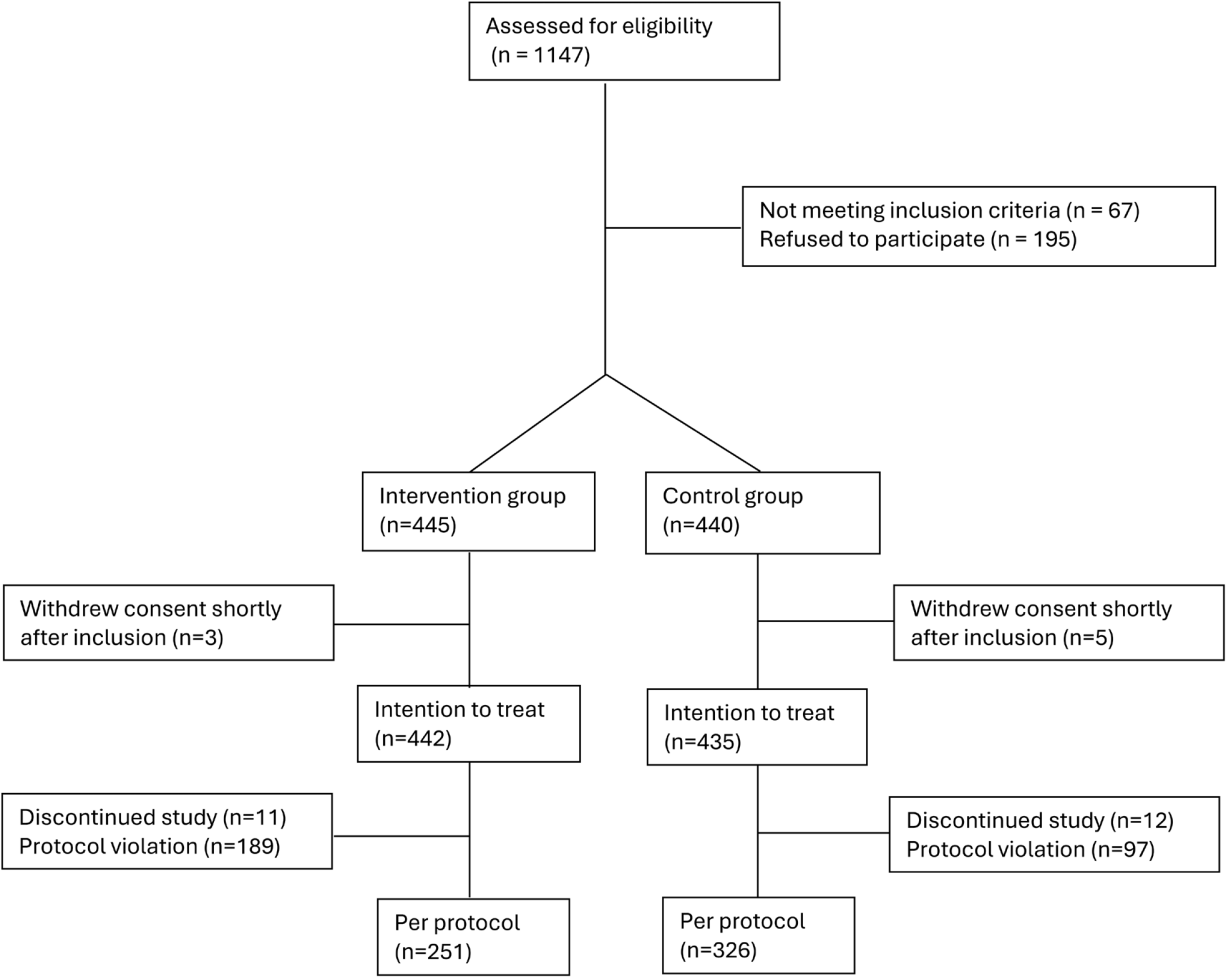
CONSORT (Consolidated Standards of Reporting Trials) flowchart recruitment of participants.

A total of 885 women with (suspected) preeclampsia were enrolled in the study between August 1, 2019, and April 30, 2024. Eight women withdrew their consent within 24 hours after inclusion, resulting in a final number of 442 and 435 in the intervention and control groups, respectively (Figure 1). In the intervention group, 11 women discontinued the study and were excluded from analysis, due to violation of >1 inclusion/exclusion criteria (n=1), loss to follow-up (n=3), withdrawal of informed consent (n=6), or the investigator’s decision (n=1). Protocol violations in the intervention group were an interval between the last PRERISK score and birth of >1 week (n=75), admitted to hospital for pregnancy-induced hypertension or (suspected) preeclampsia with a PRERISK score <5% (n=22), sent home with PRERISK score ≥5% (n=83), or having no PRERISK score measurement during pregnancy (n=9). In the control group, 12 women discontinued the study and were excluded from analysis due to loss to follow-up (n=5) and withdrawal of informed consent (n=7). Protocol violations in the control group concerned an interval between the last PRERISK score and birth of >1 week (n=97).

Baseline characteristics were similar between both groups (Table 1). The PRERISK score at inclusion was comparable (9.0% [3.8–24.2] in the intervention group versus 8.0% [3.8–20.3] in the control group) in the 2 groups.

**Table 1.**
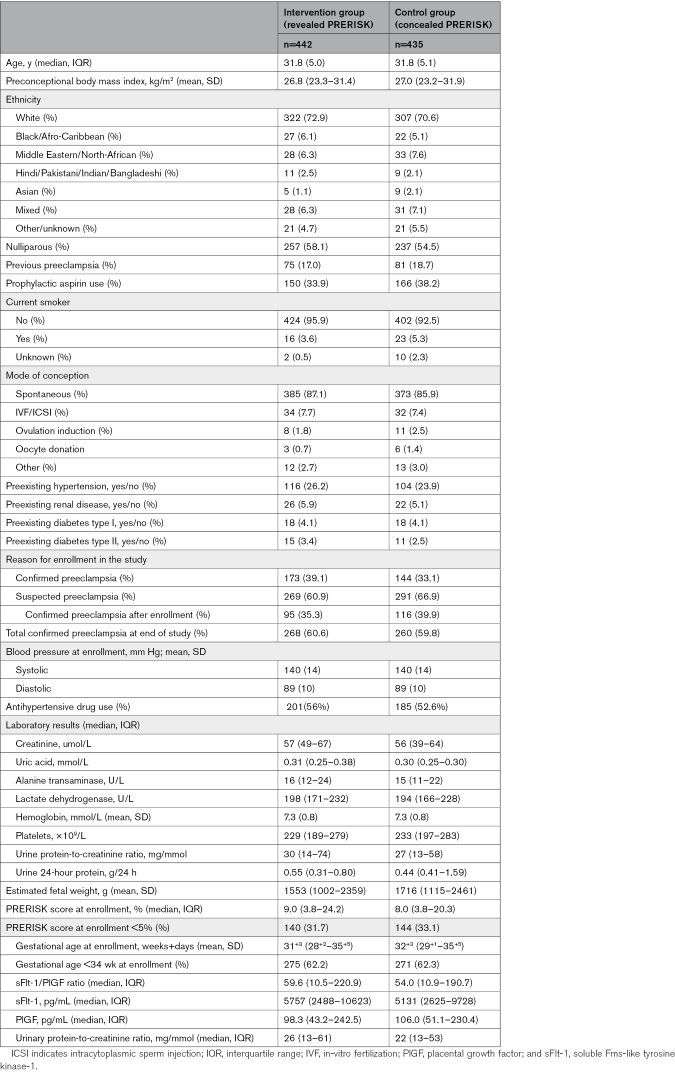
Baseline Characteristics Intention-to-Treat Population

### Noninferiority Co-Primary Outcome

In the intention-to-treat analysis, the noninferiority co-primary composite outcome of preeclampsia-related complications occurred in 41.6% of the participants in the intervention group, compared with 39.5% in the control group, with an adjusted relative risk (RR) of 1.06 ([95% CI, 0.92–1.22]; *P*=0.4254; Table 2).

**Table 2.**
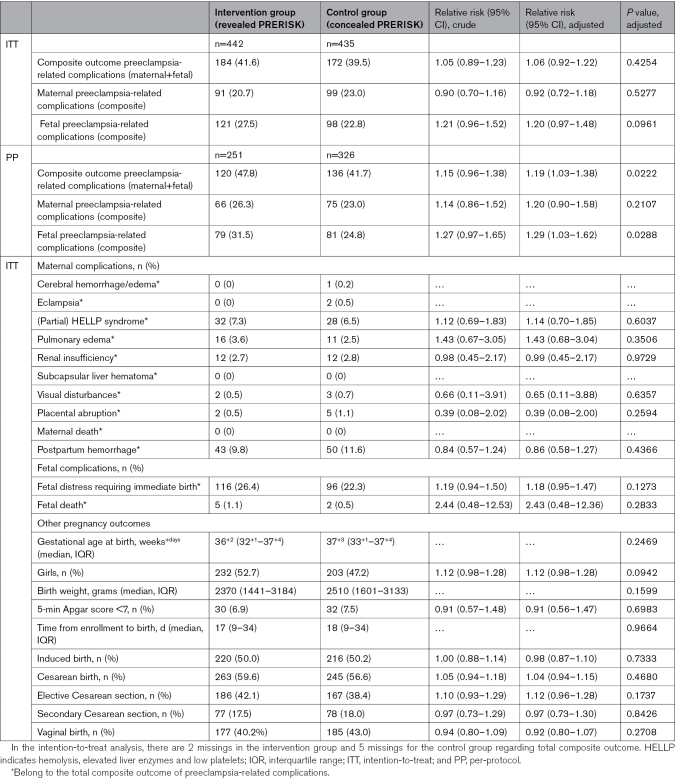
Composite Outcome Preeclampsia-Related Complications According to the ITT and PP Analysis (Co-Primary, Noninferiority Outcome)

In the per-protocol analysis, data from 251 and 326 participants in the intervention and control groups, respectively, were included (Table S3). Reasons for exclusion mainly concerned an interval between the last a PRERISK score and birth >1 week and outpatient follow-up with a PRERISK score ≥5% (Figure 1). In the per-protocol intervention group, the noninferiority co-primary composite outcome of preeclampsia-related complications occurred in 47.8% of the participants in the intervention group, compared with 41.7% in the control group (adjusted RR, 1.19 [95% CI, 1.03–1.38]; *P*=0.0222).

In the intention-to-treat analysis, noninferiority of the PRERISK score was shown. In the per-protocol analysis, criteria for noninferiority were not met (Table 2).

### Superiority Co-Primary Outcome

In the intention-to-treat analysis, the superiority co-primary outcome, that is, the proportion of women with a hospitalization ratio ≤0.05, did not differ significantly between the intervention group (23.6%) and the control group (26.3%), with an adjusted RR of 0.90 ([95% CI, 0.71–1.13]; *P*=0.3394; Table 3).

**Table 3.**
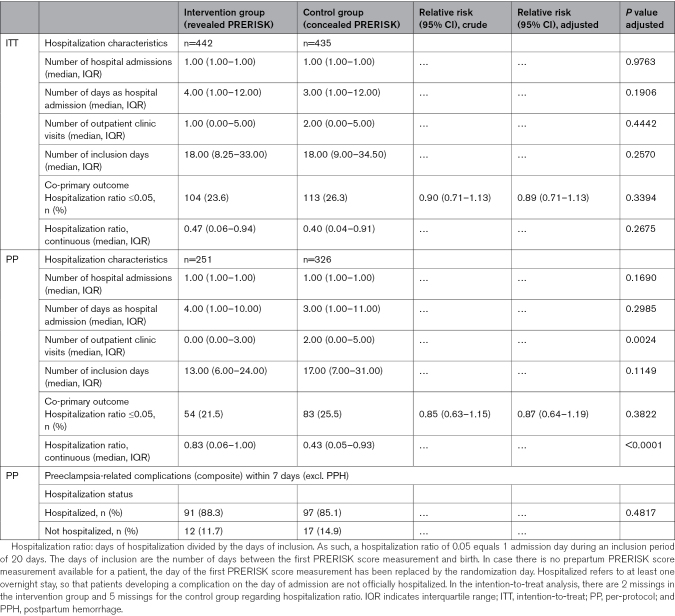
Admission Rates for the Control and Intervention Groups According to the ITT and PP Analysis (Co-Primary, Superiority Outcome)

In the per-protocol analysis, a hospitalization ratio ≤0.05 was observed in 21.5% of the participants in the intervention group, compared with 25.5% of the participants in the control group (RR, 0.87 [95% CI, 0.64–1.19]; *P*=0.3822; Table 3).

### Secondary Outcomes

Before execution of the PRERISK study and subsequent analyses, there were no subgroups identified where a larger impact of the intervention was expected. As such, no effect modification was expected and no subgroup analyses were included in the Statistical Analysis Plan. Stratification was done by gestational age and center. The stratification by center was done to equally perform randomization, and not because a difference between participating centers was expected. Comparing the groups of patients included at a gestational age <34 weeks and ≥34 weeks did not yield significant effect modification (Table S4).

When expressing the hospitalization ratio as a continuous variable, the intention-to-treat analysis revealed no difference between the groups. In the per-protocol analysis, the hospitalization ratio was significantly higher in the intervention group (0.83 [0.06–1.00] versus 0.43 [0.05–0.93]; *P*<0.001; Table 3). Among the 282 patients with a baseline PRERISK score <5%, 237 (84.0%) were discharged at study entry, while among the 538 patients with a baseline PRERISK score ≥5%, 364 (67.7%) were hospitalized at study entry.

All participants who developed a complication were already hospitalized at the time of this complication. In 12 patients in the intervention group and 17 in the control group, this complication occurred on the day of admission, that is, before an overnight stay (Table 3). Two additional sensitivity analyses were performed to correct for cases that were unjustly considered to have a hospitalization ratio of 0, due to the first PRERISK calculator measurement, admission, and delivery occurring on the same day. Outcomes were unaltered in both cases (Tables S5 and S6).

No significant difference was observed between the 2 groups for mode of birth, induction of labor, or days of inclusion (Tables 2 and 3). In the intervention group, significantly more neonates were born small-for-gestational age (46.7% versus 37.6%, *P*=0.01; Table S7), and significantly more neonates needed respiratory support (32.9% versus 26.1%, *P*=0.03; Table S7).

### Predictive Performance of PRERISK Score Compared With sFlt-1/PlGF Ratio

**Figure 2.**
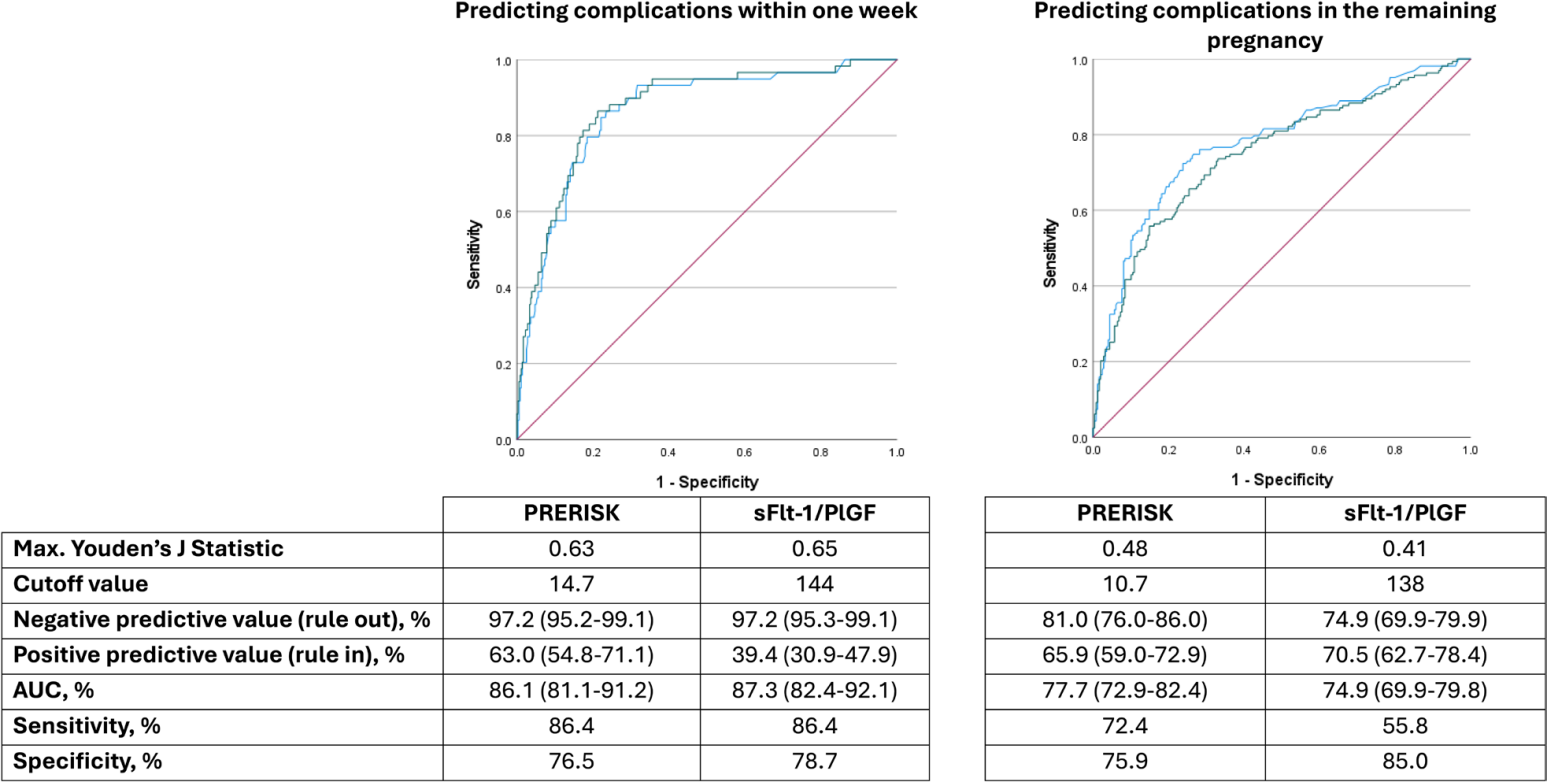
**Sensitivity and specificity (area under the curve [AUC]) for PRERISK study compared with soluble Fms-like tyrosine kinase-1 (sFlt-1)/proangiogenic placental growth factor (PlGF) ratio for composite preeclampsia-related complications within 1 week (left) and for the remaining pregnancy (right).** The blue line depicts the PRERISK score, the green line depicts the sFlt-1/PlGF ratio, the red line depicts the reference line.

Areas under the curve for the PRERISK score and the sFlt-1/PlGF ratio at baseline were determined to derive the optimum cutoff. The areas under the curves for the PRERISK score and sFlt-1/PlGF ratio to rule out a composite of preeclampsia-related complication within 1 week were 86.1% and 87.3%, respectively (Figure 2). For the PRERISK score, an optimum cutoff point of 14.7% was determined, at a maximum Youden J statistic of 0.63, resulting in a negative predictive value of 97.2% (95% CI, 95.2–99.1).

For ruling in a composite preeclampsia-related complication during the further course of pregnancy, the areas under the curves for the PRERISK score and sFlt-1/PlGF ratio were 77.7% and 74.9%, respectively. A cutoff point of 10.7% for the PRERISK score was derived, with a positive predictive value of 65.9% (95% CI, 59.0–72.9). Corresponding tables with cutoff values, sensitivity, specificity and Youden J statistic for the PRERISK score and sFlt-1/PlGF ratio predicting composite complications within 1 week or during overall pregnancy are presented in Tables S8 through S11.

Post hoc, we analyzed the duration of hospitalization using the optimal cutoff point of 14.7%, excluding all patients that were discharged with a PRERISK score <14.7%. In this selection, there were no differences at baseline between the study groups (data not shown). This analysis showed that 31.4% of patients in the intervention group had a hospitalization ratio ≤0.05, compared with 25.2% in the control group (adjusted RR, 1.21 [95% CI, 0.94–1.56]; *P*=0.14; Table S12).

### Safety

Table S13 summarizes the serious adverse events. There was no difference in maternal life-threatening events between the study groups, nor in fetal and neonatal death rates (5 fetal and 8 neonatal deaths in the intervention group [1.1% and 1.8%, respectively] versus 2 fetal and 6 neonatal deaths in the control group [0.5% and 1.4%, respectively]).

## Discussion

This randomized controlled trial showed that the application of the PRERISK score with a cutoff point of 5% in women with (suspected) preeclampsia does not reduce hospitalization. In the intention-to-treat analysis, maternal and fetal complication rates were similar in both groups, showing noninferiority.

In patients suspected of preeclampsia, the sFlt-1/PlGF ratio is a well-established biomarker for the diagnosis of preeclampsia.^13,18,20,23^ Our study has prospectively assessed the clinical impact of incorporating this ratio into a broader risk assessment tool focusing on complications. Our findings partially align with those of the PARROT (placental growth factor-based testing in women with suspected preterm pre-eclampsia) trials, which evaluated the effectiveness of PlGF-based management using an algorithm for women with suspected preeclampsia.^24,25^ An important finding of the first PARROT trial^25^ was a reduction in time-to-preeclampsia diagnosis. This was associated with a decrease in maternal complications (from 5% to 4%), without reducing infant complications. Our study assessed a broader range of maternal preeclampsia-related complications, including the development of hemolysis, elevated liver enzymes and low platelets syndrome and postpartum hemorrhage, resulting in a higher complication incidence. Unlike the PARROT trials, our study also included women with confirmed preeclampsia. This aligns with clinical practice, where confirmed preeclampsia often leads to hospitalization until birth, mostly without maternal and fetal complications. These 2 factors likely explain why we did not observe a reduction in maternal complications.

When developing the PRERISK score, we deliberately selected maternal outcomes that might require hospital admission, to reduce unnecessary hospitalization, without increasing the complication risk.^11^ Furthermore, while conducting this study, evolving guidelines recommended frequent outpatient follow-up and home monitoring rather than hospitalization with or without 24-hour urine collection. These guideline updates may have reduced the impact of the PRERISK score. Importantly, the findings of the first PARROT trial were not replicated when a similar approach was followed in Ireland, although the latter study was underpowered and included greater numbers of women with small-for-gestational-age infants at enrollment, who were otherwise normotensive.^26^ In addition, a subsequent trial investigating PlGF-based testing in a repeated manner found no improvement in maternal or perinatal outcomes,^24^ in line with our results.

In this study, we chose to implement our prediction model directly within the context of a randomized controlled trial, foregoing prior external validation. Although external validation is a key step in confirming the generalizability of the PRERISK calculator to broader populations, our decision was based on the robust internal validation already conducted.^10^ Using bootstrap resampling, the model demonstrated strong predictive performance, with minimal overfitting indicated by a shrinkage factor of 0.91. This provided sufficient confidence to move forward with a clinical trial. Furthermore, we considered that the randomized controlled trial itself, performed across multiple centers, could serve as an initial evaluation of the applicability of the PRERISK calculator in diverse clinical settings. By evaluating the model in real-world practice, we were able to observe the performance in a population beyond the initial development cohort, thus functioning as external validation in a pragmatic context.

To minimize the risk of adverse outcomes, the PRERISK score cutoff was set at 5%, based on expert consensus. When retrospectively determining the optimal cutoff value using the control group data, and subsequently analyzing the hospitalization ratio using this new cutoff (<14.7%), there was a tendency toward a reduction in hospitalization in the intervention group, which, however, still did not reach our superiority conditions.

In the per-protocol population, the intervention group displayed more cases of confirmed preeclampsia at baseline (45.4% versus 32.2%), and accordingly had a higher PRERISK score and sFlt-1/PlGF ratio, while more neonates were born small for gestational age. The most important reason for exclusion in the per-protocol analysis was outpatient follow-up despite a PRERISK score ≥5%. The relative increase in patients with more severe preeclampsia in the per-protocol intervention group after this exclusion implies that the clinical decision to send these patients home despite their PRERISK score being ≥5% was correct. Furthermore, the selective exclusion of a substantial number of patients in the intervention group may have compromised the randomized nature of the per-protocol analysis and induced confounding. When adjusting for women with confirmed preeclampsia in the per-protocol analysis, the maternal and fetal complication rates showed no difference (RR, 1.05 [95% CI, 0.89–1.24]). Hence, it is questionable whether the per-protocol analysis should be taken into account as the primary analysis regarding noninferiority. Therefore, it is likely that the PRERISK score did not influence the incidence of complications. Major strengths of our study include the unique study design, with a large sample size of women with suspected and confirmed preeclampsia, and the prospective manner in which we evaluated our sFlt-1/PlGF ratio-driven PRERISK calculator.

## Perspectives

Using the PRERISK score in patients with (suspected) preeclampsia did not show a reduction in hospitalization, while the rate of preeclampsia-related complications was similar in both groups according to intention-to-treat. Because of the increasing health care demands and costs, the implementation of a new diagnostic test should be carefully considered. Based on our findings, we do not recommend routine screening using the PRERISK calculator with a cutoff of 5% in women with suspected or confirmed preeclampsia.

## Article Information

### Acknowledgments

The authors extend their gratitude to all participants, the recruitment staff, and research nurses at each study site for their outstanding support. In addition, the authors wish to acknowledge the following individuals specifically: Erasmus MC: Ina van Gorp. Radboud UMC: Marlies Peters-Knuiman, Gerard Zijderveld, Gani Bahar. UMC Groningen: Bianca Moorman-Jager, Ruth Hiltermann-Tilanus. Amsterdam UMC: Jannet Bakker. Zuyderland Medisch Centrum: Jolanda Willems-Robberts, Brigitta Hessels-Linnemeijer. Trialbureau: Tosca van Hengel. The graphic abstract and Figure S1 were created using BioRender.

### Sources of Funding

Roche Diagnostics funded the measurement of sFlt-1 and PlGF, but had no role in the trial design, execution, data collection, analysis, or article preparation.

### Disclosures

None.

### Supplemental Material

PRERISK Investigators

Tables S1–S13

Figure S1

Original Study Protocol, Final Study Protocol and Summary of Changes

Original Statistical Analysis Plan and final Statistical Analysis Plan
